# Highly Efficient Photothermal Reduction of CO_2_ on Pd_2_Cu Dispersed TiO_2_ Photocatalyst and Operando DRIFT Spectroscopic Analysis of Reactive Intermediates

**DOI:** 10.3390/nano12030332

**Published:** 2022-01-21

**Authors:** Munirathinam Elavarasan, Willie Yang, Sethupathi Velmurugan, Jyy-Ning Chen, Thomas C.-K. Yang, Toshiyuki Yokoi

**Affiliations:** 1 Department of Chemical Engineering and Biotechnology, National Taipei University of Technology, No. 1, Section 3, Chung-Hsiao East Road, Taipei 106, Taiwan; 2011.elavarasan@gmail.com (M.E.); jingsi229@gmail.com (J.-N.C.); 2 Institute of Innovative Research, Tokyo Institute of Technology, Yokohama 226-8503, Japan; willie0106@gmail.com; 3 Precision Analysis and Material Research Center, National Taipei University of Technology, No. 1, Section 3, Chung-Hsiao East Road, Taipei 106, Taiwan; sethupathivelmurugan@gmail.com

**Keywords:** photothermal reactor, CO_2_ reduction, photocatalytic H_2_O oxidation, DRIFT, alloy

## Abstract

The photocatalytic conversion of CO_2_ to fuels using solar energy presents meaningful potential in the mitigation of global warming, solar energy conversion, and fuel production. Photothermal catalysis is one promising approach to convert chemically inert CO_2_ into value-added chemicals. Herein, we report the selective hydrogenation of CO_2_ to ethanol by Pd_2_Cu alloy dispersed TiO_2_ (P25) photocatalyst. Under UV-Vis irradiation, the Pd_2_Cu/P25 showed an efficient CO_2_ reduction photothermally at 150 °C with an ethanol production rate of 4.1 mmol g^−1^ h^−1^. Operando diffuse reflectance infrared Fourier transform (DRIFT) absorption studies were used to trace the reactive intermediates involved in CO_2_ hydrogenation in detail. Overall, the Cu provides the active sites for CO_2_ adsorption and Pd involves the oxidation of H_2_ molecule generated from P25 and C–C bond formation.

## 1. Introduction

An anthropogenic climate dramatically increases the CO_2_ level in the atmosphere, which can lead to the deterioration of the environment. The utilization of CO_2_ is an alternative route to reduce it in the environment, and it can also be converted into valuable fuels and chemicals [1,2,3,4,5]. The photocatalytic hydrogenation of CO_2_ is an efficient technique for eco-friendly conversion, but CO_2_ activation and selective hydrogenation remain a challenge [6,7]. This is due to the high thermodynamic stability and chemical inertness of CO_2_ [8,9]. Therefore, the activation and hydrogenation of CO_2_ require enormous energy to break the O=C=O bond and implement the insertion of the C-H bond. Previous research studies show that the hydrogenation of CO_2_ resulted in numerous other products. Mostly, the CO_2_ reduction processes resulted in the production of carbon monoxide (CO), a more extremely poisonous gas than CO_2_. Moreover, it is very rare to produce value added chemicals, such as methane, methanol, acids, and hydrocarbons [10,11,12,13,14,15,16,17]. Therefore, the selective hydrogenation of CO_2_ to ethanol has received great attention, and this can be achieved via electrochemical and thermochemical processes [18,19]. However, no appreciable development in the field of photo- and photothermal catalysts has been observed, which offers a sustainable method for CO_2_ to ethanol conversion.

Currently, palladium (Pd) and platinum (Pt) based catalysts are demonstrated for the selective hydrogenation of CO_2_ to ethanol [19,20,21]. In such processes, the Pd metal is cheaper than Pt, and Pd-based catalysts have worked well in the C–C coupling reactions as well [22]. Besides, the C–C coupling reaction step is the most important step to control the yield of ethanol [23]. Copper (Cu) is the cheapest and most easily available material, and it has greater affinity for CO_2_ gas. Moreover, oxides of Cu represent an efficient photocatalyst with excellent redox potential for water splitting (water oxidation) in the photocatalytic hydrogen evaluation reaction [24]. The Cu/Fe_2_O_3_ catalyst is a promising catalyst for water-splitting in thermochemical reactions, to produce H_2_ and capture CO_2_ at the same time [25]. TiO_2_ has been proven to be a most promising material in photocatalytic water splitting, pollutant degradation, self-cleaning agents, and many other fields [26,27]. The effect of reaction temperature on photodegradation performance was studied over Pd/TiO_2_ and Cu/TiO_2_ photocatalysts. The result revealed that Pd/TiO_2_ performed well during the photodegradation of methylene blue. [28] Therefore, the combination of two attractive elements is an efficient way to produce ethanol. Previous studies have demonstrated the various combinations of Pd-Cu nanoparticles that are highly selective and active catalysts for the production of ethanol from CO_2_ by thermochemical reaction [19]. Although selective hydrogenation produces valuable chemicals, the mechanism behind this selectivity is still an uncovered area. Therefore, the mechanism of selective CO_2_ reduction was probed by operando diffuse reflectance infrared Fourier transform absorption spectroscopy (DRIFTS).

The DRIFT study is used to detect the molecules and ions at their place of origin where the chemicals and substances are being produced in a chemical reaction. DRIFT analysis is a widely used technique for in-situ studies concerning the photodegradation of adsorbed dye and tracing the gas adsorbed reaction mechanism on the catalytic surface. It has also been used in agro-food industries due to its analytical compatibility with physicochemical properties of food commodities [29,30]. Moreover, DRIFT analysis was applicable to test non-transparent and indispersable materials. In our previous work, we have demonstrated photocatalytic efficiency and the reaction mechanism using DRIFT in the oxidation of ethanol [31]. The solid-state photodegradation mechanism was evaluated by DRIFT for the surface adsorbed AB-9 dye on titanium dioxide (TiO_2_) [32]. This technique was used to investigate the electron transfer and energy conversion processes at the interface, adsorbed species, and dynamics of photo-generated electrons on nitrogen-doped TiO_2_ in dye-sensitized solar cells (DSSC) [33]. Moreover, the DRIFT analysis was used to identify the reaction mechanism of CO_2_ hydrogenation [19,20,34].

In this work, we investigate the selective hydrogenation of CO_2_ to ethanol by Pd_2_Cu/P25 photocatalyst. A facile one-pot synthesis procedure was used to obtain the Pd_2_Cu alloys on TiO_2_ nanoparticles. The physicochemical properties of the as prepared Pd_2_Cu/P25 catalysts were analyzed using various analytical techniques. The hydrogenation of CO_2_ was tested using as-prepared Pd_2_Cu/P25 catalysts following the photothermal method. Moreover, the reactive intermediates and reaction mechanism of hydrogenation of CO_2_ to ethanol were determined from operando DRIFT analysis.

## 2. Materials and Methods

### 2.1. Chemicals

Palladium (II) bis(acetylacetonate) (Pd(acac)_2_), 99%), Oleylamine (70%), ethyl alcohol (99.9%), and acetone (99.9%) were purchased from Sigma Aldrich. Copper chloride dihydrate (CuCl_2_·2H_2_O, 99%), triphenylphosphine (TPP, 99%), and commercial P25 (99.9% grade) were obtained from Nacalai Tesque, Acros Organics, and Degussa, respectively. The gases deployed in this research, such as high purity nitrogen (N_2_, 99.999%), carbon dioxide (CO_2_, 99.5%), and helium (He, 99.99%), were procured from Yusheng gas company, Taipei, Taiwan.

### 2.2. Preparation of Pd_2_Cu/P25 Photocatalyst

For the synthesis of Pd_2_Cu/P25 photocatalyst, 10 mg of Pd(acac)_2_ and 55 mg of CuCl_2_·2H_2_O were dissolved in 12 mL of Oleylamine in a three-neck flask. Additionally, 90 mg of triphenylphosphine (TPP) was added. The flask was heated to 80 °C under continuous stirring in an N_2_ atmosphere for about 20 min until the chemicals were thoroughly dissolved. The temperature was then gradually raised to 170 °C, and the system was kept at this temperature for 4 h. Then, 100 mg of TiO_2_ (P25) was added to this suspension and kept for 20 min, before being naturally cooled down to room temperature. The precipitate was separated and washed with a mixture of ethyl alcohol and acetone. The obtained precipitate dried overnight at 85 °C, followed by calcination at 450 °C for 4 h.

### 2.3. DRIFT Analysis

The DRIFT experiments were conducted by Perkin Elmer FT-IR spectroscopy. The custom-designed Harrick DRIFT cell was utilized for this study. Approximately 40 mg of the photocatalyst was placed in a DRIFT cell sample holder. The sample was covered by ZnSe/CaF_2_ windows for IR radiation and a light source was passed through respectively. The DRIFT chamber was filled with N_2_ gas for 20 min to remove impurities, before the background spectrum of the sample was acquired. The temperature of the sample was controlled using a thermocouple attached to the DRIFT cell. The wavenumber resolution was fixed at 4 cm^−1^, and the experiments were carried out in the range of 500–4000 cm^−1^. For the CO_2_ reduction experiment, the CO_2_ was purged into the chamber with the water vapor at 10 cc/s for 120 min. An outlet of the reaction chamber was connected with the mass spectrum during the collection of DRIFT spectra. Meanwhile, the mass spectra wer also collected (QMS 100 Series, capillary; PEEK, and glass-lined plastic inlet type, pressure 10 mbar to 1 bar, Quadrupole type spectrometer, Faraday cup standard electron multiplier optional detector) [31].

### 2.4. Photothermal CO_2_ Reduction by the Reactor

The photothermal CO_2_ reduction was performed in a custom-made reactor. Firstly, 40 mg of Pd_2_Cu/P25 was dispersed in 50 mL of deionized (DI) water by ultrasonicator, then poured into the reactor. Therein, the magnetic stirrer continuously stirred the dispersion. Subsequently, the CO_2_ was purged into the reactor and saturated for 30 min, and then the reactor was closed perfectly. The UV-Vis (350 to 750 nm) light source (with an intensity of 380.8 μW) was used to irradiate the reactor. The reaction was kept at 80 °C by the hotplate. During each measurement, 0.1 μL of the reaction mixture was collected from the reaction and analyzed by gas chromatography (GC Model 6890 N/MS Model 5973, Agilent Technologies, Poway, CA, USA).

### 2.5. Characterizations

The crystal structures of the as-prepared samples were investigated by the X-ray diffractometer (XRD) with Cu-Kα radiation (λ = 1.5406 Å). The Raman spectra and UVDRS spectra of the samples were recorded using confocal Raman spectroscopy (ACRON Technology, Taipei, Taiwan) and UV-Vis-NIR spectroscopy (Agilent Technology, Santa Clara, CA, USA), respectively. The back scattered images of the as-synthesized samples were recorded by scanning electron microscopy (FESEM, JEOL-JSM-7610F, Tokyo, Japan). High-resolution transmission electron microscopy (HRTEM) images were obtained from JEOL TEM (JEM-2010) at 200 kV. The element composition was recorded on the energy dispersive spectrum EDS (HORIBA, X-act, Tokyo, Japan). The JEOL JPS-9030 (Tokyo, Japan) X-ray photoelectron spectrometer (XPS) was deployed to analyze the surface properties of the prepared samples. The dissolved solution of the as prepared photocatalyst was analyzed by the inductively coupled plasma-atomic emission spectrometer (ICP-AES, Shimadzu ICPE-9000, Kyoto, Japan) for the quantitative elemental contents in each sample. 

### 2.6. Dark Adsorption by CO_2_ with Transmission FTIR

The dark adsorption experiment was carried out in the transmission mode by a spectrometer (JASCO FT-IR 4600, Tokyo, Japan) equipped with a Mercury Cadmium Telluride detector. The sample was pressed into a self-supporting disk (20 mm diameter, ca. 30 mg) and placed in an IR cell attached to a gas-phase closed circulatory system for observation. The sample was pretreated at 723 K for 1 h under vacuum and then probed by a fixed amount of CO_2_ vapor from 5 to 500 Pa at ambient temperature. After the dark adsorption at 500 Pa, the system was evacuated to vacuum again to remove free CO_2_ and other free carbonyl species, to investigate the chemically adsorbed CO_2_ species on the samples.

## 3. Results and Discussion

### 3.1. Material Characterizations

**Figure 1:**
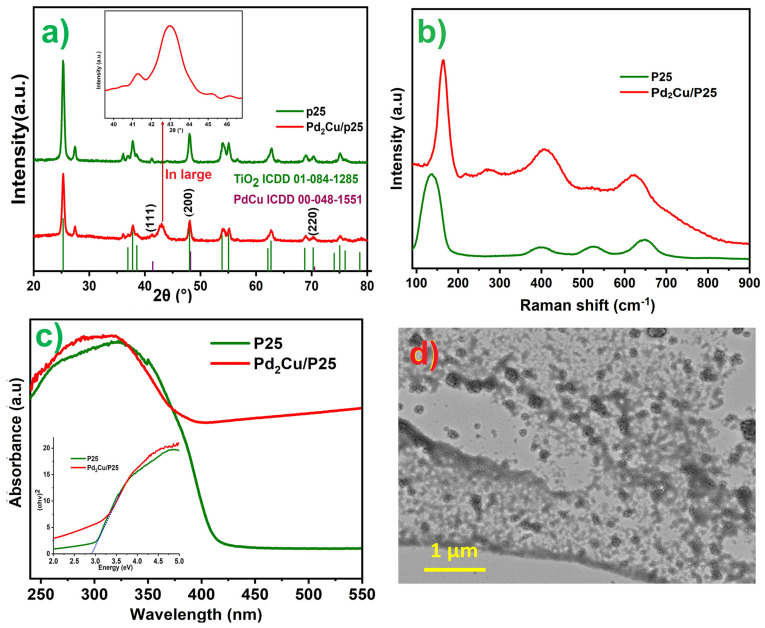
(**a**) XRD pattern, (**b**) Raman spectra (**c**) UV−Vis (DRS), inset is showing Tauc plots of P25 and Pd_2_Cu/P25, (**d**) FESEM Backscattered−Electron (BSE) Image of Pd_2_Cu/P25.

Figure 1a shows the X-ray diffraction patterns of Pd_2_Cu alloy on P25 along with the diffraction pattern of pristine P25. The observed crystalline peaks at 41.11, 48.46, and 71.02° are attributed to the (111), (200), and (220) planes of PdCu alloy that are matched with the ICDD No. 00-048-1551 [35]. The obtained crystalline parameters indicate that the Pd_2_Cu alloy is crystallized in the body-centered cubic crystal. The crystal structure of Pd_2_Cu is illustrated in the inset of Figure 1a.

Moreover, the remaining peaks at 25.38, 38.23, 48.47, 54.71, 56.01, 27.58, 36.90, 41.60, and 55.0° are assigned to the (101), (004), (200), (105), (211), (110), (101), (210), and (211) planes of TiO_2_ (P25) respectively [31]. It can be seen from XRD peaks that the intensities of Pd_2_Cu peaks are dominated by the P25 crystalline peaks. This is because of the low loading level and high dispersion of Pd_2_Cu on P25. Figure 1b shows the Raman spectra of Pd_2_Cu/P25 and P25. The typical peaks appearing at 142, 397, 533, and 651 cm^−1^ correspond to the Eg, B1g, A1g, and Eg modes of TiO_2_ (P25), respectively. The additional peaks that appeared at 218 and 279 cm^−1^ are correlated to the specific interactions between PdCu alloys and P25. The PdCu alloys have no vibrations. However, the corresponding oxides of Pd and Cu revealed significant peaks. The vibrations at 398 and 650 cm^−1^ are increased for the Pd_2_Cu/P25 when compared with P25. This might be attributed to the stretching vibrations of Pd-O and Cu-O. Moreover, the light absorption properties of Pd_2_Cu/P25 were investigated by UV-Vis spectra. As shown in Figure 1c, the UV-visible absorption spectra of Pd_2_Cu/P25 exhibited stronger absorption in the visible range when compared to the P25. The indirect bandgap of Pd_2_Cu/P25 is estimated from the plots of (αhv)^1/2^ vs. hv (energy of light), which shows 3.0 eV for both Pd_2_Cu/P25 and P25 (Figure 1c, inset). Furthermore, the FESEM backscattered electron (BSE) image gives the topography of the P25 supported Pd_2_Cu alloys.

**Figure 2:**
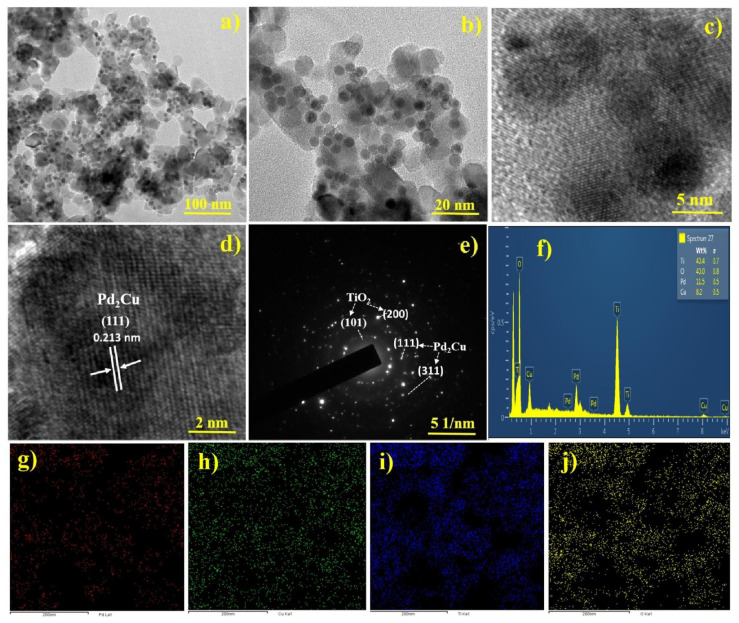
(**a**–**c**) low and high magnified TEM images, (**d**) lattice fringes, (**e**) SAED patterns, (**f**) EDX spectra, (**g**–**j**) mapping of Pd_2_Cu/P25 (Elements: Pd (**g**); Cu (**h**); Ti (**i**); O (**j**)).

Figure 1d shows the BSE image of Pd_2_Cu/P25, wherein the Pd_2_Cu alloy shows a stronger backscatter electron signal, which can be seen as dark spots. P25 exhibits a weaker electron signal. This BSE image can help to distinguish the Pd_2_Cu alloy and P25 in the highly dispersed Pd_2_Cu/P25 catalyst. Figure 2a displays the TEM images of Pd_2_Cu alloy on the P25 nanoparticles, wherein the 5 ± 1 nm-sized Pd_2_Cu nanodots are highly dispersed on 20 ± 5 nm-sized P25 nanoparticles. The high magnification images (Figure 2b,c) clearly show the mono dispersion of Pd_2_Cu nanodots and their lattice fringes (Figure 2d). Figure 2e shows the SAED patterns of Pd_2_Cu/P25, which are taken at a random angle to view clear ED patterns. The obtained ring patterns are indexed to the (101), (200), and (111), (311) planes, which are in accordance with the XRD patterns of P25 and Pd_2_Cu, respectively. The uniform distribution and the ratio of elemental percentage are investigated using the EDX and mapping studies. Figure 2f reveals 82% of P25 and 18% of Pd_2_Cu alloy in the photocatalyst, respectively. Moreover, elemental mapping of Pd_2_Cu/P25 was performed and the results are shown in Figure 2g–j. Thus, it confirmed that the Pd_2_Cu nanodots are uniformly distributed on the P25 nanoparticles, supporting better photothermal CO_2_ conversion.

**Figure 3:**
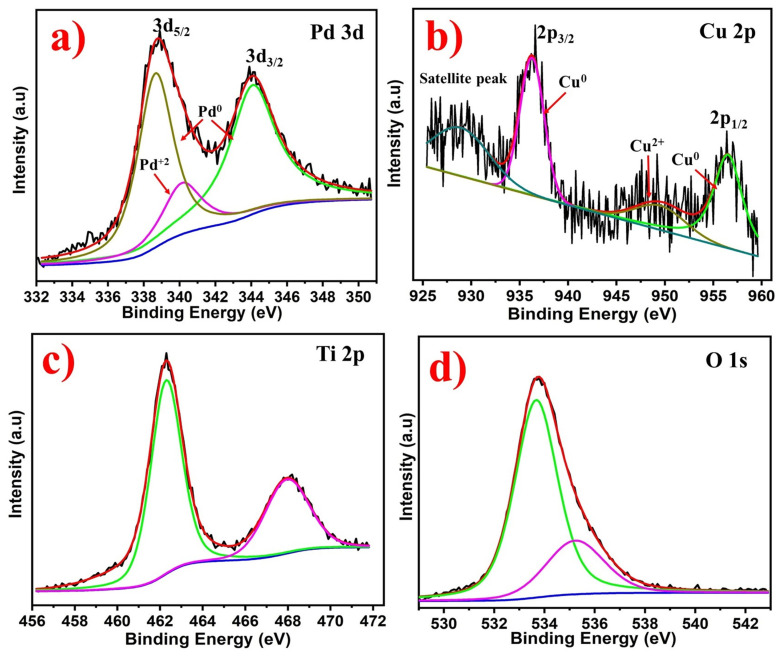
(**a**) Pd 3d, (**b**) Cu 2p, (**c**) Ti 2p, and (**d**) O 1s XPS spectra of Pd_2_Cu/P25.

The X-ray photoelectron spectra (XPS) were used to explore the surface properties of Pd_2_Cu/P25. The narrow scan Pd 3d spectrum shows two peaks for spin-orbits 3d_5/2_ and 3d_3/2_ at 339 and 345 eV, corresponding to the metallic Pd^0^ respectively (Figure 3a). Moreover, the deconvolution spectra of Pd 3d show an additional peak for the Pd^2+^, which is attributed to PdO on the surface [36]. Figure 3b shows the narrow scan spectrum of Cu 2p. The binding energies at 937 and 957 eV correspond to the 2p_3/2_ and 2p_1/2_ spin-orbits, respectively. The Cu 2p spectrum was deconvoluted into four peaks. The two main peaks denote Cu in the metallic state, while the minor peaks denote Cu in +2 oxidation states. Similar to Pd, Cu also exhibited CuO on its surface. Moreover, the peak intensity of Cu 2p was lower than that of Pd, which is in accordance with EDX results. Additionally, Figure 3c,d shows the narrow scan peaks of Ti 2p and O 1s, which are indexed to the typical peaks of TiO_2_ (P25). The Ti 2p spectra shows two peaks at the binding energies of 462.1 and 467.8 eV belonging to the 2p_3/2_ and 2p_1/2_ orbitals, respectively. The deconvoluted O 1s peak reveals two binding energies at 533.5 and 535.3 eV, corresponding to the surface hydroxyl groups and lattice oxygen atoms respectively. Overall, these characterizations confirm the formation of Pd_2_Cu alloy on the P25 support. The amount of metals proportion in the photocatalyst were further confirmed by ICP-AES. For the analysis, the powder of the as-prepared catalyst was soaked in the aqua regia solutions for 6 hours and then mixed with 30wt% HF for overnight. The results were summarized in Table 1.

### 3.2. Photothermal CO_2_ Reduction

**Figure 4:**
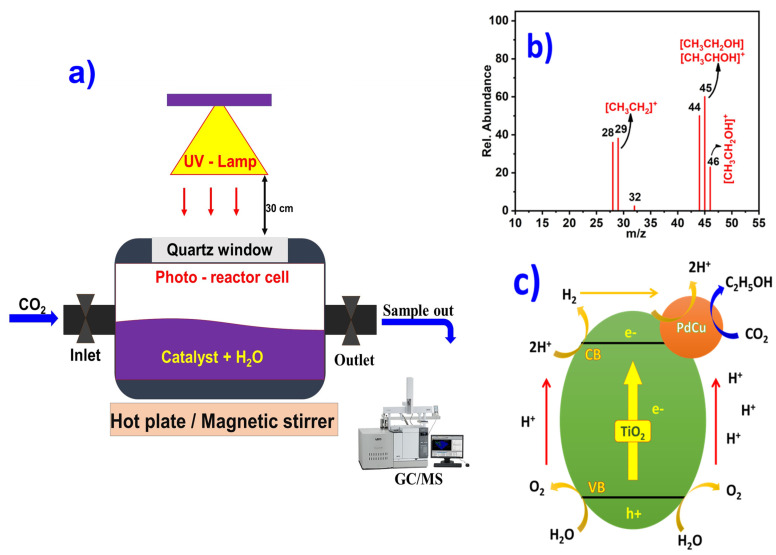
(**a**) Schematic diagram of photothermal CO_2_ reduction using photoreactor, (**b**) GCMS spectrum of ethanol after 1h UV-Vis light irradiation at 80 °C. (**c**) The overall process of CO_2_ reduction and H_2_O oxidation on Pd_2_Cu/P25 (CB: conduction band, VB: valence band, h^+^: holes, and e^−^: electrons).

The photothermal hydrogenation of CO_2_ over a Pd_2_Cu/P25 catalyst was evaluated using the photoreactor. The detailed experimental procedure is presented in the experimental section and the schematic diagram of the reactor setup is shown in Figure 4a. The reaction was continued for up to six hours, while the sample was taken from the reaction mixture and subjected to the GCMS analysis. In the first hour, no products were seen in the reaction mixture. Afterwards, the GCMS shows the peaks for ethanol, as shown in Figure 4b. The typical fragmentation of mass 45 and 46 m/z correspond to the [C_2_H_5_O]^+^ and [C_2_H_5_OH]^+^ respectively [37]. The ethanol yield is shown in Table 2, and the yield was compared with other reported catalysts. The photocatalytic CO_2_ reduction of Pd_2_Cu/P25 was also compared with pristine P25. The P25 showed no signal for ethanol production. Herein, the P25 (TiO_2_) oxidized the water at its valence band, and the electrons were utilized by Pd_2_Cu at the conduction band. Further, the Pd_2_Cu has reduced the CO_2_ by capturing the H^+^ from aqueous media. Therefore, without Pd_2_Cu, it is difficult to produce ethanol by P25 alone. Figure 4c shows the schematic representation of the overall reaction process. Furthermore, the reaction mechanism of CO_2_ reduction and the intermediate products were explored by the DRIFT analysis.

### 3.3. The FTIR Spectra of the Dark Adsorption of CO_2_ on Photocatalysts

**Figure 5:**
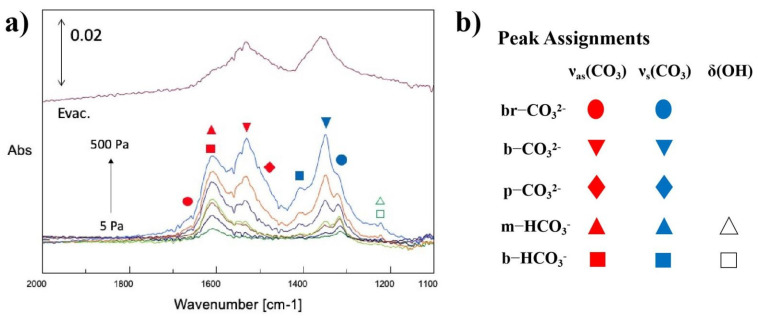
(**a**) The FTIR spectra of the dark adsorption on the Pd_2_Cu/P25 photocatalysts and (**b**) peak assignments at various pressure gradients from 5 Pa to 500 Pa of CO_2_ vapor and finally in vacuum condition.

An airtight gas-cell FTIR accessory with 15 cm pathlength for transmission mode measurements was used to analyze the extent of interactions between adsorbates and photocatalysts. Figure 5 illustrates the FTIR spectra of Pd_2_Cu/P25 probed by CO_2_ from 5 to 500 Pa at ambient temperature. After the CO_2_ adsorption at 500 Pa was completed, the system was evacuated to vacuum again to remove the free CO_2_ and free carbonate species. The evacuated spectrum is symbolized as “Evac.” in Figure 5. This information provides strong evidence of the formation of chemical adsorption bonding between our sample and carbonate species. The peaks are assigned following the reference [43,44,45,46,47]. These spectra feature two major species, namely carbonates and bicarbonates, that were adsorbed on the catalysts by a few different kinds of connections. Starting with bicarbonates, the band at 1215 cm^−1^ is related to the (OH) vibrational signals of monodentate and bidentate bicarbonates (m-HCO_3_^−^ and b-HCO_3_^−^). The band at around 1625 cm^−1^ can be ascribed to the asymmetric vibrational signals (v_as_(CO_3_)) of both monodentate and bidentate bicarbonates (m-HCO_3_^−^ and b-HCO_3_^−^) [43,44,45,46]. However, the significant bands at 1425 cm^−1^, identified as the symmetric vibration signal (_vs_(CO_3_)) of the bidentate bicarbonates (b-HCO_3_^−^), implies that the bicarbonate groups on the Pd_2_Cu/P25 were dominated by bidentate bicarbonates (b-HCO_3_^−^).

The bands related to carbonate species were also investigated. The broad bands at 1675 cm^−1^ and 1325 cm^−1^ represented the asymmetric vibrational signals (v_as_(CO_3_)) and the symmetric vibration signal (v_s_(CO_3_)) of the adsorbed bridged carbonates (br-CO_3_^2−^), respectively [43,44,45,46]. The significant bands at 1525 cm^−1^ and 1350 cm^−1^ exhibited the asymmetric vibrational signals (v_as_ (CO_3_)) and the symmetric vibration signal (v_s_(CO_3_)) of the adsorbed bidentate carbonates (b-CO_3_^2−^), respectively. Besides, a small shoulder at 1500 cm^−1^ (asymmetric vibrational signals (v_as_ (CO_3_)) indicated that there was a trace amount of poly-dentate carbonate species (p-CO_3_^2−^) observed by IR. After the sample reached equilibrium at 500 Pa of CO_2_, the system was then turned in to vacuum for 30 min. As shown in Figure 5, the remaining signals mostly belonged to carbonates. This result can provide further insights in DRIFT analysis when the reaction was applied.

Thus, the dark adsorption FTIR analysis probed by CO_2_ was carried out using Pd_2_Cu/P25. This analysis gave us a closer look at the micro-environment around the active sites and the adsorbent, which helped us rationalize the photocatalytic performance of each sample in the next section.

### 3.4. In-Situ DRIFT Investigation of Photothermal CO_2_ Reduction

**Figure 6:**
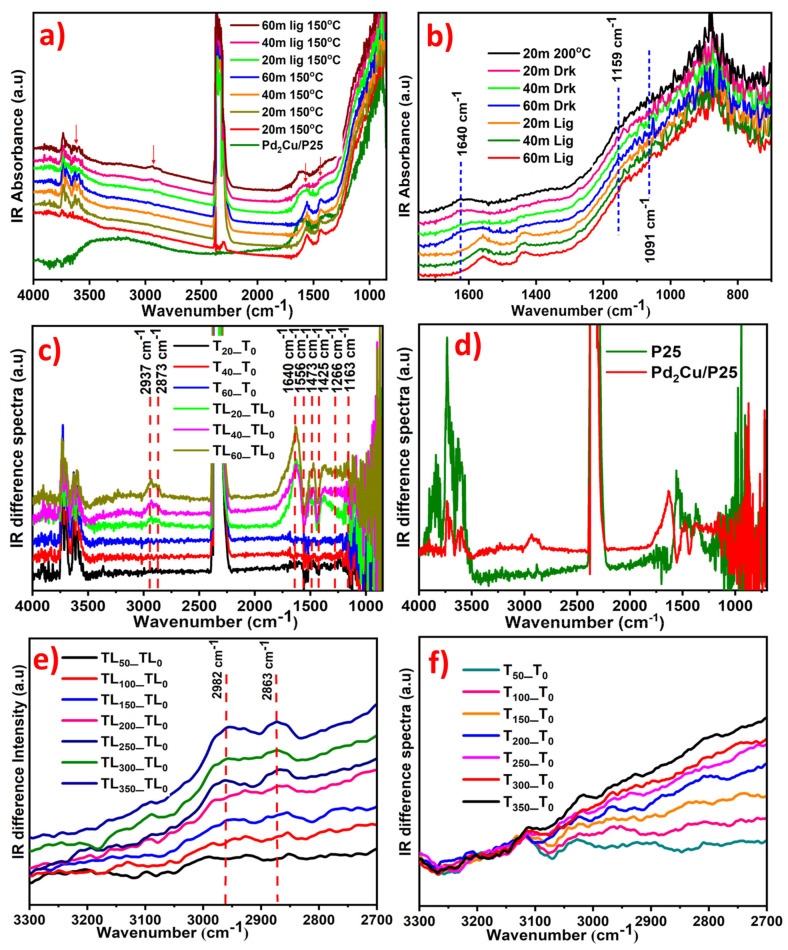
(**a**,**b**) Time-dependent DRIFT spectra of Pd_2_Cu/P25 and (**c**) IR difference spectra of CO_2_ reduction on Pd_2_Cu/P25. (**d**) IR difference spectra of P25 and Pd_2_Cu/P25 after 1 h light irradiation. DRIFT spectra CO_2_ reduction on Pd_2_Cu/P25 at various temperatures (**e**) with and (**f**) without light irradiation.

The mechanisms of CO_2_ reduction and resultant intermediate products were monitored by in-situ DRIFT analysis. Figure 6a shows the time-dependent DRIFT absorbance spectra of Pd_2_Cu/P25. The pristine Pd_2_Cu/P25 shows peaks of around 3500–3000 cm^−1^ and 1320 cm^−1^ for the surface hydroxyl group (moisture). These surface hydroxyl groups were initially removed by thermal treatment at 150 °C for 20 m in a nitrogen atmosphere. After removing the moisture, the CO_2_ and gas-phase H_2_O were passed into the drift cell at 150 °C. The spectra were collected in time intervals (t) of 20, 40, and 60 min in darkness (absence of light) and in the presence of light. The peaks appearing at 2346 cm^−1^ and 3700–3500 cm^−1^ correspond to the ʋ_3_ and ʋ_3_ + ʋ_1_ stretching vibrations of adsorbed CO_2_, respectively. Additionally, the monodentate carbonate CO_3_^2−^ peak appeared at 1085 cm^−1^ for the metal carbonate (m-C–O–) [18], which confirmed the CO_2_ adsorption on Pd_2_Cu/P25 in dark.

This metal carbonate (m-C–O–) peak and the CO_2_ peaks (3700–3500 cm^−1^) were slowly decreased when irradiated with UV-Vis light, and at the same time, the C–H peaks are increased at 2962 cm^−1^ (Figure 6a). In addition, the *CO peak arises at 1640 cm^−1^ during the light irradiation, as shown in Figure 6b [19]. The obtained DRIFT spectra were derived to visualize the significant changes. Thereby, the obtained IR spectra were subtracted from the initial absorbance spectra. The T_0_, T2_0_, T4_0_, and T_60_ refer to the spectra taken in darkness at t = 20, 40, and 60 min respectively. Similarly, TL_0_, TL_20_, TL_40_, and TL_60_ refer to the spectra under UV-Vis light irradiation at t = 20, 40, and 60 min respectively. As shown in Figure 6c, the specific peaks at 2937, 2873 cm^−1^ (asymmetric and symmetric C-H stretching frequency), and 3250 cm^−1^ (–OH symmetric stretching) are increased with the reaction time [48,49,50]. The strong peaks at 1640 and 1473 cm^−1^ were also increased, which correspond to the *CO and CH_2_ (scissoring), respectively. The small peaks at 1266 and 1163 cm^−1^ refer to the formation of C–C (stretching frequency) and CO_2_^δ−^, respectively [19]. The peaks at 1556 and 1425 cm^−1^ correspond to the CO_3_^2−^ and CHCOH respectively. These two peaks are gradually decreased by further increasing the reaction time. From these observations, we have illustrated the mechanistic pathway of CO_2_ reduction given below.

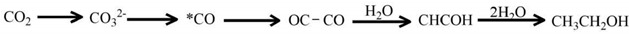



In the above reaction steps, the CO_2_ gas-phase is adsorbed as a monodentate metal carbonate (m-C–O–) CO_3_^2−^. On the catalytic (Cu) surface, the CO_3_^2−^ to *CO conversion occurs via π–π interaction by the excited electrons [46]. The local concentration of CO on the catalyst (Pd) undergoes the C–C coupling step, and then the OC–CO reacts with water and produces the intermediates of CHCOH. Furthermore, the addition of water with CHCOH produces the final product of ethanol [18]. Overall, DRIFT studies have demonstrated the reaction pathways wherein Cu was assumed as a CO_2_ adsorption site. It is very difficult to separate the active sites in DRIFT spectra where the CO_2_ is adsorbed. However, previous studies have demonstrated that CO_2_ adsorption is more favorable on the Cu site [25].

On other hand, Pd was engaged in the C–C coupling reaction, as well as the highly selective hydrogenation of CO_2_ to C_2_H_5_OH. The production of ethanol was confirmed by the mass spectra. The CO_2_ reduction on Pd_2_Cu/P25 was compared with P25. The corresponding DRIFT results are shown in Figure 6d. Hence, the Pd_2_Cu/P25 is a more selective and active catalyst that produces ethanol when compared to P25. Moreover, the photothermal hydrogenation of CO_2_ over the Pd_2_Cu/P25 catalyst was evaluated at various temperatures under UV-Vis light irradiation (Figure 6e). The DRIFT difference spectra reveal that the C_2_H_5_OH formation (identified from C-H peaks arising) increased with an increase in temperature. In contrast, the reaction was continued without light irradiation at 150 °C, exhibiting no C–H peak, and with no ethanol formation in dark (Figure 6f). Therefore, the light provides the driving force for CO_2_ reduction (0.611 V) and water oxidation (1.23 V). Overall, the reaction begins with the oxidation of water on P25 and then the electrons that evolved during water oxidation are utilized by Pd_2_Cu. The H_2_ molecules are generated from the P25 as a result of photocatalytic water oxidation. The Cu provides the active site for CO_2_ adsorption and Pd oxidizes the generated H_2_ molecules and involves the C–C bond formation. The key step of the CO_2_ reduction is the hydrogenation of *CO to *HCO, which is performed by the Pd active sites.

## 4. Conclusions

This work reports the photothermal reduction of CO_2_ and the oxidation of H_2_O over the Pd_2_Cu/P25 photocatalyst. The formation of ethanol was confirmed from GCMS and online mass spectroscopy. The Pd_2_Cu/P25 showed an efficient CO_2_ reduction photothermally at 150 °C with an ethanol production rate of 4.1 mmol g^−^^1^ h^−^^1^ under UV-Vis irradiation. Other products were not visible on GCMS spectra due to their low quantity. However, operando DRIFT absorption spectroscopy traces the intermediate products. The mechanism of CO_2_ reduction on the Pd_2_Cu/P25 catalyst was monitored by DRIFTS. The reaction begins with the oxidation of water on P25 and then the electrons involved in water oxidation were utilized by Pd_2_Cu. The Pd active sites utilize those electrons and hydrogenate the *CO to *HCO, which is a key step for the reduction of CO_2_ to ethanol. Moreover, the Pd_2_Cu/P25 catalyst demonstrated the highly active and selective hydrogenation of CO_2_ to C_2_H_5_OH.

## Figures and Tables

**Table 1:** ICP-AES results of Pd_2_Cu/P25 photocatalyst.

ICP Analysis	Pd	Cu	Ti
mg L^−1^	0.0416	0.0129	0.232
Molecular Weight	106.42	63.54	47.867
mMolL^−1^	3.9 × 10^−4^	2.0 × 10^−4^	4.8 × 10^−3^
Molar ratios	~2	1	24

**Table 2:** The comparison of ethanol yield on Pd_2_Cu/P25 with various catalysts.

Catalysts	Gas (CO_x_)	Temperature (°C)	C_2_H_5_OH Yields (mmol g^−1^ h^−1^)	Reference
1% Pt/Co_3_O_4_	CO_2_	200	0.07	[38]
1.2% RhMnLiFe	CO	320	3.5	[39]
Rh/SiO_2_	CO	250	0.37	[40]
Au/TiO_2_	CO_2_	200	2.82	[41]
8.7% Pd-10% Cu/SiO_2_	CO_2_	250	1.12	[42]
Pd_2_Cu/P25	CO_2_	150	4.1	This work

## Data Availability

Data are contained within the article.

## References

[1] Yoon Y., Hall A.S., Surendranath Y. (2016). Tuning of silver catalyst mesostructure promotes selective carbon dioxide conversion into fuels. Angew. Chem. Int. Ed..

[2] Rodemerck U., Holeňa M., Wagner E., Smejkal Q., Barkschat A., Baerns M. (2013). Catalyst development for CO_2_ hydrogenation to fuels. ChemCatChem.

[3] Behrens M. (2016). Promoting the Synthesis of Methanol: Understanding the requirements for an industrial catalyst for the conversion of CO_2_. Angew. Chem. Int. Ed..

[4] Behrens M. (2014). Heterogeneous catalysis of CO_2_ conversion to methanol on copper surfaces, Angew. Chem. Int. Ed..

[5] Robatjazi H., Zhao H., Swearer D.F., Hogan N.J., Zhou L., Alabastri A., McClain M.J., Nordlander P., Halas N.J. (2017). Plasmon-induced selective carbon dioxide conversion on earth-abundant aluminum–cuprous oxide antenna-reactor nanoparticles. Nat. Commun..

[6] Wang W., Wang S., Ma X., Gong J. (2011). Recent advances in catalytic hydrogenation of carbon dioxide. Chem. Soc. Rev..

[7] Klankermayer J., Wesselbaum S., Beydoun K., Leitner W. (2016). Selective catalytic synthesis using the combination of carbon dioxide and hydrogen: Catalytic chess at the interface of energy and chemistry. Angew. Chem. Int. Ed..

[8] Lu Q., Rosen J., Zhou Y., Hutchings G.S., Kimmel Y.C., Chen J.G., Jiao F. (2014). A selective and efficient electrocatalyst for carbon dioxide reduction. Nat. Commun..

[9] Kwak J.H., Kovarik L., Szanyi J. (2013). Photocatalytic reduction of CO_2_ on TiO_2_ and other semiconductors, CO_2_ reduction on supported Ru/Al_2_O_3_ catalysts: Cluster size dependence of product selectivity. ACS Catal..

[10] Porosoff M.D., Yang X., Boscoboinik J.A., Chen J.G. (2014). Molybdenum carbide as alternative catalysts to precious metals for highly selective reduction of CO_2_ to CO. Angew. Chem. Int. Ed..

[11] Martin O., Martín A.J., Mondelli C., Mitchell S., Segawa T.F., Hauert R., Drouilly C., Curulla-Ferré D., Pérez-Ramírez J. (2016). Indium oxide as a superior catalyst for methanol synthesis by CO_2_ hydrogenation. Angew. Chem. Int. Ed..

[12] Studt F., Sharafutdinov I., Abild-Pedersen F., Elkjær C.F., Hummelshøj J.S., Dah S., Chorkendorff I., Nørskov J.K. (2014). Discovery of a Ni-Ga catalyst for carbon dioxide reduction to methanol. Nat. Chem..

[13] Graciani J., Mudiyanselage K., Xu F., Baber A.E., Evans J., Senanayake S.D., Stacchiola D.J., Liu P., Hrbek J., Sanz J.F. (2014). Highly active copper-ceria and copper-ceria-titania catalysts for methanol synthesis from CO_2_. Science.

[14] Moret S., Dyson P.J., Laurenczy G. (2014). Direct synthesis of formic acid from carbon dioxide by hydrogenation in acidic media. Nat. Commun..

[15] Zhu Y., Zhang S., Ye Y., Zhang X., Wang L., Zhu W., Cheng F., Tao F. (2012). Catalytic conversion of carbon dioxide to methane on Ruthenium-Cobalt bimetallic nanocatalysts and correlation between surface chemistry of catalysts under reaction conditions and catalytic performances. ACS Catal..

[16] Mistry H., Varela A.S., Bonifacio C.S., Zegkinoglou I., Sinev I., Choi Y.-W., Kisslinger K., Stach E.A., Yang J.C., Strasser P. (2016). Highly selective plasma-activated copper catalysts for carbon dioxide reduction to ethylene. Nat. Commun..

[17] Choi Y.H., Jang Y.J., Park H., Kimb W.Y., Lee Y.H., Choi S.H., Lee J.S. (2017). Carbon dioxide Fischer–Tropsch synthesis: A new path to carbon-neutral fuels. Appl. Catal. B Environ..

[18] Li F., Li Y.C., Wang Z., Li J., Nam D.-H., Lum Y., Luo M., Wang X., Ozden A., Hung S.-F. (2020). Cooperative CO_2_-to-ethanol conversion via enriched intermediates at molecule–metal catalyst interfaces. Nat. Catal..

[19] Bai S., Shao Q., Wang P., Dai Q., Wang X., Huang X. (2017). Highly Active and Selective Hydrogenation of CO_2_ to Ethanol by Ordered Pd−Cu Nanoparticles. J. Am. Chem. Soc..

[20] Qian C., Sun W., Hung D.L.H., Qiu C., Makarem M., Kumar S.G.H., Wan L., Ghoussoub M., Wood T.E., Xia M. (2019). Catalytic CO_2_ reduction by palladium-decorated silicon–hydride nanosheets. Nat. Catal..

[21] Fujitani T., Nakamura I. (2002). Methanol Synthesis from CO and CO_2_ Hydrogenations over Supported Palladium Catalysts. Bull. Chem. Soc. Jpn..

[22] Crudden C.M., Sateesh M., Lewis R. (2005). Mercaptopropyl-modified mesoporous silica: Remarkable support for the preparation of a reusable, heterogeneous palladium catalyst for coupling reactions. J. Am. Chem. Soc..

[23] Pang S.H., Schoenbaum C.A., Schwartz D.K., Medlin J.W. (2013). Directing reaction pathways by catalyst active-site selection using self-assembled monolayers. Nat. Commun..

[24] Lee G.-J., Anandan S., Masten S.J., Wu J.J. (2016). Photocatalytic hydrogen evolution from water splitting using Cu doped ZnS microspheres under visible light irradiation. Renew. Energy.

[25] Imtiaz Q., Yüzbasi N.S., Abdala P.M., Kierzkowska A.M., Beek W.V., Brodaa M., Müller C.R. (2016). Development of MgAl_2_O_4_-stabilized, Cu-doped, Fe_2_O_3_-based oxygen carriers for thermochemical water-splitting. J. Mater. Chem. A..

[26] Hunge Y.M., Yadav A.A., Mathe V.L. (2019). Photocatalytic hydrogen production using TiO_2_ nanogranules prepared by hydrothermal route. Chem. Phys. Lett..

[27] Gosavi S., Tabei R., Roy N., Latthe S.S., Hunge Y.M., Suzuki N., Kondo T., Yuasa M., Teshima K., Fujishima A. (2021). Low Temperature Deposition of TiO_2_ Thin Films through Atmospheric Pressure Plasma Jet Processing. Catalysts.

[28] Chen Y.-W., Hsu Y.-H. (2021). Effects of reaction temperature on the photocatalytic activity of TiO_2_ with Pd and Cu cocatalysts. Catalysts.

[29] Lohumi S., Lee S., Lee H., Cho B.K. (2015). A review of vibrational spectroscopic techniques for the detection of food authenticity and adulteration. Trends Food Sci. Technol..

[30] Manju G., Archana J., Verma K.K. (2022). Dispersive liquid–liquid microextraction and diffuse reflectance-Fourier transform infrared spectroscopy for iodate determination in food grade salt and food samples. Food Chem..

[31] Elavarasan M., Uma K., Yang T.C.-K. (2019). Photocatalytic oxidation of ethanol using ultrasonic modified TiO_2_; an in-situ diffuse reflectance infrared spectroscopy study. Results Phys..

[32] Yang T.C.-K., Wang S.-F., Tsai S.H.-Y., Lin S.-Y. (2001). Intrinsic photocatalytic oxidation of the dye adsorbed on TiO_2_ photocatalysts by diffuse reflectance infrared Fourier transform spectroscopy. Appl. Catal. B Environ..

[33] Liu J., Winwarid P., Yang T.C.-K., Chuang S.S.-C. (2018). In situ infrared study of photo-generated electrons and adsorbed species on nitrogen-doped TiO_2_ in dye-sensitized solar cells. Phys. Chem. Chem. Phys..

[34] Xu F., Meng K., Cheng B., Yu J., Ho W. (2019). Enhanced Photocatalytic Activity and Selectivity for CO_2_ Reduction over a TiO_2_ Nanofiber Mat Using Ag and MgO as Bi-Cocatalyst. ChemCatChem..

[35] Yang Q., Le X., Li Y., Ian T.M., Guo F., Tao M., Yang R., Qi L., Lin Z., Gu S. (2018). BCC-Phased PdCu Alloy as a Highly Active Electrocatalyst for Hydrogen Oxidation in Alkaline Electrolytes. J. Am. Chem. Soc..

[36] Gu Z., Xiong Z., Ren F., Li S., Xu H., Yan B., Du Y. (2018). Flower-like PdCu catalyst with high electrocatalytic properties for ethylene glycol oxidation. J. Taiwan Inst. Chem. E.

[37] Tiscione N.B., Alford I., Yeatman D.T., Shan X. (2011). Ethanol Analysis by Headspace Gas Chromatography with Simultaneous Flame-Ionization and Mass Spectrometry Detection. J. Anal. Toxicol..

[38] He Z., Qian Q., Ma J., Meng Q., Zhou H., Song J., Liu Z., Han B. (2016). Water-enhanced synthesis of higher alcohols from CO_2_ hydrogenation over a Pt/Co_3_O_4_ catalyst under Milder conditions, Angew. Chem. Int. Ed..

[39] Pan X., Fan Z., Chen W., Ding Y., Luo H., Bao X. (2007). Enhanced ethanol production inside carbon-nanotube reactors containing catalytic particles. Nat. Mater..

[40] Yang N., Medford A.J., Liu X., Studt F., Bligaard T., Bent S.F., Nørskov J.K. (2016). Intrinsic selectivity and structure sensitivity of rhodium catalysts for C_2+_ oxygenate production. J. Am. Chem. Soc..

[41] Wang D., Bi Q., Yin G., Zhao W., Huang F., Xie X., Jiang M. (2016). Direct synthesis of ethanol via CO_2_ hydrogenation using supported gold catalysts. Chem. Commun..

[42] Jiang X., Koizumi N., Guo X., Song C. (2015). Bimetallic Pd–Cu catalysts for selective CO_2_ hydrogenation to methanol. Appl. Catal. B Environ..

[43] Baltrusaitis J., Jensen J.H., Grassian V.H. (2006). FTIR spectroscopy combined with isotope labeling and quantum chemical calculations to investigate adsorbed bicarbonate formation following reaction of carbon dioxide with surface hydroxyl groups on Fe_2_O_3_ and Al_2_O_3_. J. Phys. Chem. B.

[44] Turek A.M., Wachs I.E., DeCanio E. (1992). Acidic properties of alumina-supported metal oxide catalysts: An infrared spectroscopy study. J. Phys. Chem. B.

[45] Collins S.E., Baltanás M.A., Bonivardi A.L. (2006). Infrared spectroscopic study of the carbon dioxide adsorption on the surface of Ga_2_O_3_ polymorphs. J. Phys. Chem. B.

[46] Collins S.E., Baltanas M.A., Bonivardi A.L. (2004). An infrared study of the intermediates of methanol synthesis from carbon dioxide over Pd/β-Ga_2_O_3_. J. Catal..

[47] Köck E.M., Kogler M., Bielz T., Klötzer B., Penner S. (2013). In situ FT-IR spectroscopic study of CO_2_ and CO adsorption on Y_2_O_3_, ZrO_2_, and yttria-stabilized ZrO_2_. J. Phys. Chem. C.

[48] Yu Z., Chuang S.S.C. (2007). In situ IR study of adsorbed species and photo-generated electrons during photocatalytic oxidation of ethanol on TiO_2_. J. Catal..

[49] Elavarasan M., Uma K., Yang T.C.-K. (2021). Nanocubes phase adaptation of In_2_O_3_/TiO_2_ heterojunction photocatalysts for the dye degradation and tracing of adsorbed species during photo-oxidation of ethanol. J. Taiwan Inst. Chem. E.

[50] Guzman F., Chuang S.S.C. (2010). Tracing the Reaction Steps Involving Oxygen and IR Observable Species in Ethanol Photocatalytic Oxidation on TiO_2_. J. Am. Chem. Soc..

